# Sensitivity analyses for data missing at random versus missing not at random using latent growth modelling: a practical guide for randomised controlled trials

**DOI:** 10.1186/s12874-022-01727-1

**Published:** 2022-09-24

**Authors:** Andreas Staudt, Jennis Freyer-Adam, Till Ittermann, Christian Meyer, Gallus Bischof, Ulrich John, Sophie Baumann

**Affiliations:** 1 Department of Methods in Community Medicine, Institute of Community Medicine, University Medicine Greifswald, Walther-Rathenau-Str. 48, 17475 Greifswald, Germany; 2 Institute and Policlinic of Occupational and Social Medicine, Faculty of Medicine, TU Dresden, Fetscherstr. 74, 01307 Dresden, Germany; 3 Institute for Medical Psychology, University Medicine Greifswald, Walther-Rathenau-Str. 48, 17475 Greifswald, Germany; 4 German Centre for Cardiovascular Research (DZHK), Partner site Greifswald, Fleischmannstr. 8, 17475 Greifswald, Germany; 5 Department SHIP-KEF, Institute of Community Medicine, University Medicine Greifswald, Walther-Rathenau-Str. 48, 17475 Greifswald, Germany; 6 Department of Prevention Research and Social Medicine, Institute of Community Medicine, University Medicine Greifswald, Walther-Rathenau-Str. 48, 17475 Greifswald, Germany; 7 Department of Psychiatry and Psychotherapy, University of Lübeck, Ratzeburger Allee 160, 23538 Lübeck, Germany

**Keywords:** MAR, MNAR, Growth curve model, Participant attrition, Dropout

## Abstract

**Background:**

Missing data are ubiquitous in randomised controlled trials. Although sensitivity analyses for different missing data mechanisms (missing at random vs. missing not at random) are widely recommended, they are rarely conducted in practice. The aim of the present study was to demonstrate sensitivity analyses for different assumptions regarding the missing data mechanism for randomised controlled trials using latent growth modelling (LGM).

**Methods:**

Data from a randomised controlled brief alcohol intervention trial was used. The sample included 1646 adults (56% female; *mean age* = 31.0 years) from the general population who had received up to three individualized alcohol feedback letters or assessment-only. Follow-up interviews were conducted after 12 and 36 months via telephone. The main outcome for the analysis was change in alcohol use over time. A three-step LGM approach was used. First, evidence about the process that generated the missing data was accumulated by analysing the extent of missing values in both study conditions, missing data patterns, and baseline variables that predicted participation in the two follow-up assessments using logistic regression. Second, growth models were calculated to analyse intervention effects over time. These models assumed that data were missing at random and applied full-information maximum likelihood estimation. Third, the findings were safeguarded by incorporating model components to account for the possibility that data were missing not at random. For that purpose, Diggle-Kenward selection, Wu-Carroll shared parameter and pattern mixture models were implemented.

**Results:**

Although the true data generating process remained unknown, the evidence was unequivocal: both the intervention and control group reduced their alcohol use over time, but no significant group differences emerged. There was no clear evidence for intervention efficacy, neither in the growth models that assumed the missing data to be at random nor those that assumed the missing data to be not at random.

**Conclusion:**

The illustrated approach allows the assessment of how sensitive conclusions about the efficacy of an intervention are to different assumptions regarding the missing data mechanism. For researchers familiar with LGM, it is a valuable statistical supplement to safeguard their findings against the possibility of nonignorable missingness.

**Trial registration:**

The PRINT trial was prospectively registered at the German Clinical Trials Register (DRKS00014274, date of registration: 12th March 2018).

## Background

Participant attrition is common in longitudinal intervention studies [1, 2], especially those targeting substance use behaviour such as alcohol consumption [3, 4]. The reasons for dropout or non-participation in follow-ups can be manifold. In this context, three missing data mechanisms are usually distinguished [5, 6]: missing completely at random (MCAR), missing at random (MAR) or missing not at random (MNAR). Missing data can be considered MCAR if the missingness does not depend on any observed or unobserved information and is therefore truly random, for instance when data loss occurs unsystematically due to technical errors. In the case of MAR, the missingness depends on and can be sufficiently explained by observed variables such as sociodemographic characteristics or information from previous assessments. By contrast, MNAR must be assumed if the missingness is systematically associated with the unobserved data itself, for instance when participants in behaviour change intervention trials who did not benefit from the intervention are less likely to participate in follow-ups than those who did benefit.

Despite missing data being ubiquitous, missing data mechanisms often are not examined rigorously [7–10], probably due to a lack of an easy to implement missing data strategy. Rather, deficient ad-hoc strategies such as complete case analysis or single imputation methods are applied frequently [9, 11]. Neglecting missing data mechanisms may lead to power reduction, biased statistical inference, and invalid conclusions about an intervention’s efficacy [12–17]. Due to its strict requirements (propensity for missing data is completely unrelated to observed and unobserved variables), MCAR is only rarely met in empirical research. Besides, there are no statistical tests to disentangle if missing data are MAR or MNAR [18]. Assuming MAR does seem reasonable when retention and dropout can be predicted by observed variables. However, MNAR can never be ruled out completely. As an example, some participants in a randomised controlled trial assigned to an alcohol intervention may reduce their alcohol use over the course of the study, while others may maintain or even increase their alcohol use. If one of those groups is less willing to participate in follow-up assessments, MNAR seems equally reasonable. Since the distinction between MAR and MNAR involves considerable uncertainty, sensitivity analyses are indicated [19, 20]. The goal would be to compare results under different assumptions about the causes of the missing data (MAR vs. MNAR).

Latent growth modelling (LGM) offers a flexible framework in which it is possible to incorporate missing values under MAR as well as MNAR assumptions [21, 22]. With LGM, inter-individual differences (e.g. intervention vs. control group) in the intra-individual development over time can be analysed [23]. Individual trajectories, measured by a repeated outcome variable, are captured by latent growth factors (intercept and slope). In recent years, LGM has been increasingly used for the evaluation of randomised controlled trials, e.g. targeting alcohol use [24–27]. Usually, LGMs are fit to the data using a full-information maximum likelihood estimator (FIML) assuming the missing data to be MAR. What is more, LGM can also be estimated under a MNAR assumption [21].

Growth models that assume the missing data to be MNAR can be divided into selection [28], shared parameter [29] and pattern mixture models [30]. These MNAR models integrate model components to account for the process that generated the missing data [21]. Selection and shared parameter models complement LGMs by regressions to predict the missingness of the outcome. In order to do this, binary missing data indicators (0 = observed, 1 = missing) are linked to the growth model via logistic regression equations. In the model proposed by Diggle and Kenward [28], missing data indicators are directly regressed on the repeated outcome measure. By doing this, the missingness becomes dependent on the unobserved values themselves, thus modelling a non-ignorable dropout process. It should be noted that selection models (originating back to Heckman [31]) are not specific to LGM but a generic approach to incorporate MNAR processes in multivariate statistical models. The approach by Wu and Carroll [29] is quite similar whereas the missing data indicators are regressed on the individual growth trajectories. This means that the missingness becomes dependent on the rate of change over time, including the entirety of observed and unobserved values on the repeated outcome variable. Hence, the propensity of a repeated outcome to be missing at time point *t* either depends on that same outcome at time point *t* and *t-1* (Diggle-Kenward model) or on the latent intercept and slope factors (Wu-Carroll model). Pattern mixture models take a different approach and divide the sample into subgroups that share the same (or similar) missing data patterns [21]. Each pattern is defined by a combination of observed and missing values on the repeated outcome variable (e.g. intermittent missing data, permanent dropout). With pattern mixture models, the LGM is estimated separately for each subgroup allowing for differential growth trajectories in the predefined groups. Parameter estimates for the whole sample are obtained by calculating the weighted average of the growth model parameters of each subgroup. With the pattern mixture approach, the missingness is therefore not used as an outcome, but as a predictor to inform the stratification of the sample into distinct groups. Readers interested in the technical details of the models described above may be directed to seminal work in this area [6, 32].

All MNAR growth models have one crucial limitation: they depend on untestable assumptions to achieve model identification. The Diggle-Kenward model assumes a multivariate normal distribution of the repeated outcome measure, the Wu-Carroll model a multivariate normal distribution of the shared parameters, i.e. the latent intercepts and slopes. For pattern mixture models, identifying parameter restrictions are necessary. Different possibilities exist (complete case restriction, neighbouring case restriction, and available case restriction) to constrain inestimable parameters from one subgroup to the same parameter from one or more of the other subgroups where that parameter can be estimated. Since the underlying assumptions cannot be tested and parameter estimates may be biased in the presence of violations of these assumptions [21], it is inadvisable to base conclusions on MNAR models only. Rather, sensitivity analyses are warranted to examine if the conclusions drawn from a particular study differ, depending on whether an ignorable (MAR) or non-ignorable (MNAR) missing data mechanism is assumed [20, 33]. Assessing how sensitive results are to different missing data mechanisms is widely recommended [17, 20, 21, 33–35], but only rarely realised [7, 8, 36].

The aim of the present study was to demonstrate the use of LGM as a means to evaluate randomised controlled trials under MAR vs. MNAR assumptions. For this purpose, data from the PRINT (“Testing a proactive expert system intervention to prevent and to quit at-risk alcohol use”) trial was used, a randomised controlled trial comparing a brief alcohol feedback intervention to assessment only. By doing this in a non-technical and readily accessible manner, we provide a practical guide to conduct sensitivity analyses for different missing data mechanisms to answer the question: How do conclusions about intervention efficacy change if one alters the assumptions about the process that resulted in missing data?

## Methods

### PRINT trial

The PRINT trial was a two-armed, parallel group randomised controlled trial to examine the efficacy of computer-generated individualised alcohol feedback among a general population sample of 1,646 adults with past year alcohol consumption. The sample was recruited between April and July 2018 in the waiting area of the local registration office in Greifswald, Mecklenburg-Western Pomerania, Germany. All trial participants provided written informed consent. Details on the recruitment procedure, inclusion criteria, sample description, and primary outcome results have been published elsewhere [37]. The PRINT trial was prospectively registered at the German Clinical Trials Register (DRKS00014274, date of registration: 12/03/2018) and approved by the ethics committee of the University Medicine Greifswald, Germany (protocol number BB 147/15). The protocol was published on 9 July 2018 [38]. Additional follow-ups to investigate long-term intervention effects were approved by the ethics committees of University Medicine Greifswald (protocol number BB 053/19) and TU Dresden (protocol number SR-EK-272062020).

Participants were randomised to the intervention or control group by the tablet computers using a computer-generated list of random numbers (simple randomisation with 1:1 allocation ratio). The participants remained blinded to their group assignment until they received the intervention or not. Study assistants, responsible for eligibility screening and recruitment, were blinded to the participants’ group assignment. Intervention group participants received three intervention letters by mail at baseline, 3 and 6 months later. The letters to the study participants were generated by a computer expert system. It automatically selected feedback components for the letters to the study participants based on pre-defined decision rules. For the letters to be individualised and tailored to the participants’ personal situation, self-report data was collected beforehand via computer-assisted telephone interviews. The data was used by the expert system to compose the feedback letters that were then sent to the participants via mail. The control group did not receive any feedback. To control for the effect of the repeated assessments, the same self-report data was collected at the same time points from control group participants.

Study assistants conducted computer-assisted telephone interviews 3 (t1), 6 (t2), 12 (t3), and 36 (t4) months after baseline (t0). The assessments were identical for both the intervention and control group and covered self-reported alcohol consumption as well as psychological variables regarding the motivation to change one’s alcohol use. At each time point, questionnaires were sent out per mail or e-mail after 10 unsuccessful contact attempts via telephone. Participants received up to three vouchers worth 5 Euro in compensation, one directly after giving their consent to participate, one prior to t3, and one after completion of the follow-up assessment at t4.

### Measures

For the present study, the sum score of the *Alcohol Use Disorders Identification Test – Consumption* (AUDIT-C; [39]) was used as outcome measure. It ranges between 0 and 12 and is calculated from three questions asking for the typical frequency of alcohol use, the typical amount of alcohol consumed when drinking, and the typical frequency of heavy episodic drinking (4 or more alcoholic drinks on one occasion for women and 5 or more for men). Higher AUDIT-C sum scores indicate higher alcohol consumption. The AUDIT-C score was also used to distinguish low-risk and at-risk alcohol use, based on sex-specific cut-off values (≥ 4 for women and ≥ 5 for men) [40].

Auxiliary variables, i.e. further information used to estimate missingness, encompassed self-reported sex, age, school education, living together with a partner (yes / no), self-reported health, and smoking (response options: never, former, occasional, or daily smokers). For school education, the highest general educational degree was assessed and condensed into a binary variable (less than 12 vs. 12 or more years of school education). Self-reported health in general (based on [41]) was rated on a 5-point Likert scale (1 = excellent, 2 = very good, 3 = good, 4 = fair, 5 = poor).

### Statistical analysis

The dataset and syntax supporting the conclusions of this article are available via the Research Data Centre at Leibniz Institute for Psychology (ZPID) and can be accessed via 10.5160/psychdata.stas21pr11. The analysis can be divided into three main steps (Table 1). In Step 1, information about the potential missing data mechanism was gathered. In Step 2, unadjusted and adjusted growth models were calculated to analyse intervention effects over time that assumed the missing data to be MAR. In Step 3, the findings from Step 2 were safeguarded by incorporating MNAR mechanisms into the growth model.

**Table 1:** Three-step approach to sensitivity analyses for data missing at random versus missing not at random in randomised controlled trials

	What to do	What to get
Step 1: Missing data patterns and mechanisms	- Determine the percentage of missing data at each time point - Examine missing data patterns (e.g. plotting the outcome for each missing data pattern) - Predict participation in follow-ups using baseline characteristics (e.g. logistic regression) - Use other available information (e.g. process data)	Evidence to support assumptions about the process that lead to missing data (MAR versus MNAR)
Step 2: MAR models	- Determine the best-fitting shape of growth in preliminary models - Calculate unadjusted growth model regressing the latent growth factors on the participants’ group assignment - Add covariates to the model	Evidence about intervention efficacy under the assumption that data are missing at random
Step 3: MNAR models	- Generate missing data indicators - Predict missing data indicators by outcome at time point *t* and the previous time point *t-1* (Diggle-Kenward model) - Predict missing data indicators by latent growth factors (Wu-Carroll model) - Calculate the growth model for different subgroups that share the same missing data pattern (pattern mixture model) - Compare the results to determine the sensitivity of the conclusions for different assumptions regarding the missing data	Evidence about intervention efficacy under the assumption that data are missing not at random

*MAR* Missing at random, *MNAR* Missing not at random

#### Step 1: Missing data patterns and mechanisms

To begin with, evidence for the possible data generating process was gathered. The percentage of missing data at each measurement point was determined and compared between the two study conditions. Since all participants provided baseline data, and with four measurement points after baseline, 16 missing data patterns were possible and analysed descriptively. For each missing data pattern, the average AUDIT-C sum scores over time were plotted for the intervention and control group. Prior work on reach and retention suggested that dropout during the active intervention phase (t0 – t2) was associated with age, school education and smoking [38]. Two logistic regression models predicting participation at t3 and t4 respectively, were conducted to complement these findings. Sex, age, school education, living together with a partner, study condition, self-reported health, smoking status, and alcohol-related risk level at baseline were used as predictors. Significant predictors would support the plausibility of the MAR assumption. All analyses from Step 1 were conducted with Stata 14 [42].

#### Step 2: MAR models

**Fig. 1:**
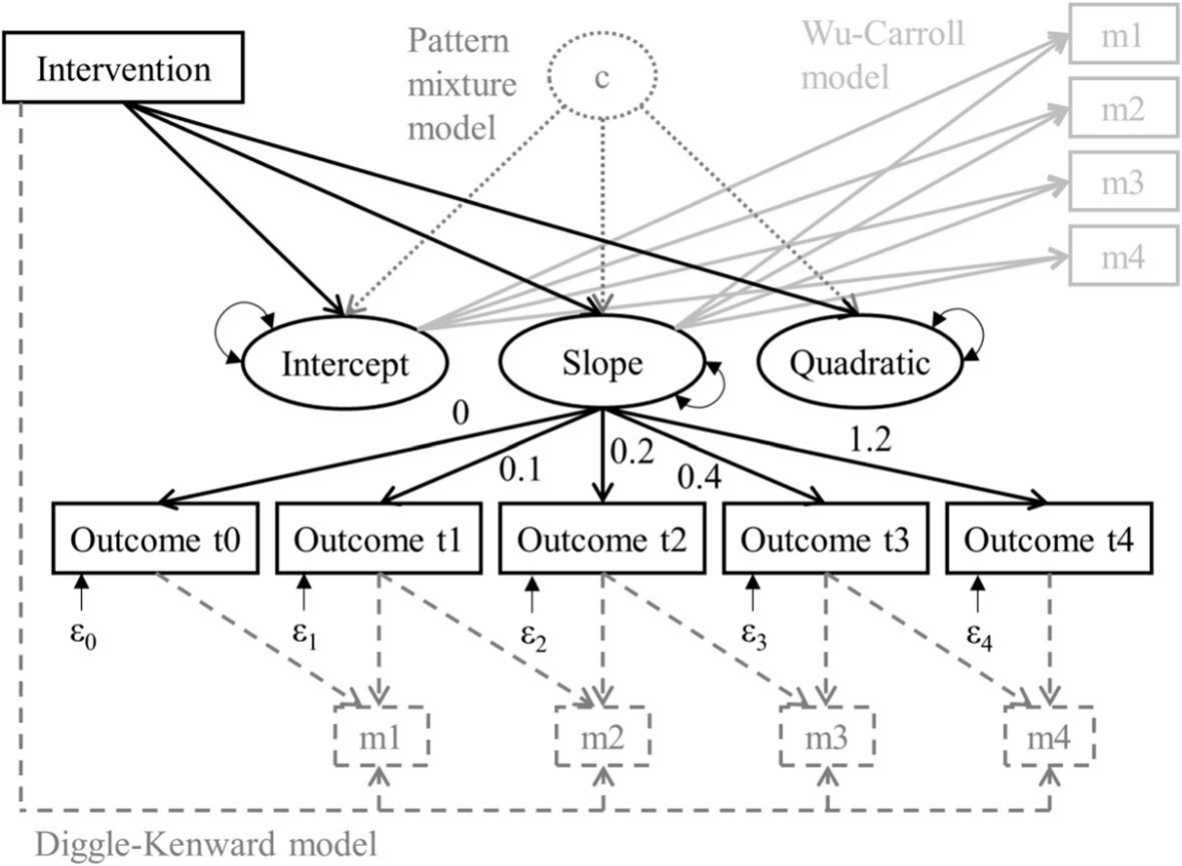
Latent growth model assuming missing data to be MAR (solid black) to calculate differences between intervention and control group, amended by three MNAR sensitivity analyses: Diggle-Kenward selection model (dashed grey), Wu-Carroll shared parameter model (solid grey) and pattern mixture models (dotted grey). *Note*. The factor loadings from the latent slope factor to the repeated outcome variables correspond to the time between measurement occasions (0.1 = 3 months). The arrows and factor loadings for the latent intercept and quadratic factor were omitted for clarity. ε_0_ to ε_4_ represent residual variances of the repeated outcome measures. m1 to m4 represent missing data indicators indicating for every participant if the outcome at the respective time point t1 to t4 was observed (m = 1) or not (m = 0)

LGM was applied in Mplus version 7.31 [43] to evaluate the efficacy of the intervention after 12 and 36 months. The AUDIT-C sum score was used as repeated outcome and manifest indicator of growth over time, captured by latent growth factors (solid black in Fig. 1). Preliminary analyses were conducted to determine the shape of growth over time. For that purpose, unconditional LGMs with different sets of growth factors were calculated (Mplus syntax 1a-1c). Factor loadings for the latent slope factor were set at 0, 0.1, 0.2, 0.4, and 1.2 and represented the time between measurement occasions (0.1 = 3 months). Accordingly, the loadings for the latent quadratic factor were set at 0, 0.01, 0.04, 0.16, and 1.44 (omitted in Fig. 1 for clarity). Models were compared using the Bayesian Information Criterion (BIC; [44]), the Comparative Fit Index (CFI) and the Root Mean Square Error of Approximation (RMSEA). As all three models had CFIs > 0.98 and RMSEA < 0.08, the decision was based on BIC. This information criterion balances fit and parsimony, where lower BIC values indicate better fitting models. Comparisons revealed that a model with intercept, linear and quadratic slope provided the best fit to the data (Mplus syntax 1b).

Then, the latent growth factors were regressed on the participants’ group assignment (0 = control group, 1 = intervention group). That allowed to calculate group differences at t3 and t4, as well as the difference in the change of the AUDIT-C score over time between intervention and control group (see section MODEL CONSTRAINTS in Mplus syntax 2a). These differences were given as absolute numbers with 95% confidence intervals (*95% CI*). The model (solid black in Fig. 1) was estimated under an MAR assumption using a full-information maximum likelihood estimator (FIML) with robust standard errors. With FIML, all available data in the variance-covariance matrix are used to find the model parameter values that maximize the likelihood for the observed data. FIML has been shown to produce accurate and unbiased estimates when data are truly missing at random [12].

Next, an adjusted LGM was calculated (not included in Fig. 1 to avoid visual clutter). Covariates were included to support the plausibility of the MAR assumption, i.e. the missingness can be sufficiently explained by observed (baseline) variables. Therefore, sex, age, school education, living together with a partner, self-reported health, smoking, and alcohol-related risk level observed at t0 were added as covariates (Mplus syntax 2b). To prevent a non-positive latent variable covariance matrix, the variance of the quadratic growth factor was fixed to zero for this model. Since the unadjusted growth model (CFI = 0.995; RMSEA = 0.039) fit the data better than the adjusted model (CFI = 0.952; RMSEA = 0.086), all consecutive sensitivity analyses were built upon the model without covariates. However, calculating the following MNAR models with prognostic covariates may be worthwhile to increase the power of the analysis in some cases [45]. On the other hand, the addition of covariates might impede successful model convergence.

#### Step 3: MNAR models

In Step 3, different growth models were estimated that assumed the missing data to be MNAR. By comparing the findings from the MAR (Step 2) and MNAR models (Step 3), it was possible to examine how sensitive the results were for different assumptions about the missing data mechanism. For the Diggle-Kenward selection model ([28]; dashed grey in Fig. 1), the growth model was amended by a set of logistic regression equations, through which the propensity for each time point *t* to be missing was predicted by the outcome at time point *t* and the outcome observed at the previous time point (*t-1*). For that purpose, four missing data indicators (m1 – m4) were generated, indicating for each participant if the AUDIT-C score was observed (m = 1) or not (m = 0). Two coding schemes for the missing indicators can be distinguished: the survival indicator coding scheme and the multinomial coding scheme (Table 2) depending on the assumed data generating process. In general, longitudinal studies can feature sporadically missing (intermittent) or permanently missing values (dropout). In the case of survival indicators, only permanent attrition is assumed to be caused by a MNAR mechanism, whereas intermittently missing values are assumed to be MAR [21]. In contrast, the multinomial coding scheme differentiates and allows to predict both, intermittent and permanent missingness.

**Table 2:** Missing data indicator coding schemes

Survival indicator coding scheme	Multinomial coding scheme
0 = observed value or intermittent missing (reference)	0 = intermittent missing
1 = permanent dropout	1 = permanent dropout
99 = dropout at previous time point	2 = observed value (reference)
	99 = dropout at previous time point

Since one can only speculate about the true data generating process, it is advisable to examine if the choice of coding scheme affects the model results. Therefore, two Diggle-Kenward models were calculated, one with survival indicators (Mplus syntax 3a) and one with multinomial indicators of missingness (Mplus syntax 3b). To generate the survival missing indicators, the Mplus function SDROPOUT in the DATA MISSING command part can be used. The multinomial missing indicators had to be recoded from the missing data patterns using the Mplus DEFINE command. Recall that the binary (Mplus syntax 3a) and multinomial logistic regressions (Mplus syntax 3b) predicting the propensity of an outcome to be missing are supposed to account for the data to be MNAR. These equations can only be solved because a multivariate normal distribution of the repeated outcome variables is assumed [28]. Group differences were calculated in the same way as in the previous models using the MODEL CONSTRAINT command.

For the Wu-Carroll shared parameter model ([29]; solid grey in Fig. 1), the propensity for an outcome variable to be missing is predicted by the overall growth trajectory over time, i.e. the missing data indicators (m1 – m4) are regressed on the latent growth factors. As with the Diggle-Kenward model, both coding schemes for the missing indicators were implemented in two separate models (Mplus syntax 3c and 3d). In our case, the Wu-Carroll model did not converge when the missing data indicators were regressed on all three latent growth factors (intercept, linear, and quadratic). Therefore, the models were adapted, and the quadratic growth factor was omitted from the logistic regression. Mplus still produced a warning (mismatch between observed and expected information matrices), suggesting that the estimated standard errors may not be trustworthy. Using the MLF instead of the MLR estimator provided a remedy and ultimately led to a successful and reliable convergence of the Wu-Carroll models.

For the pattern mixture models (dotted grey in Fig. 1), multiple group analysis was used, in which the latent growth model was estimated separately for different subgroups that shared the same missing data pattern. In our case, estimating the growth model in 16 distinct subgroups was not feasible. Therefore, subgroups of participants with similar missing data patterns were assembled. This decision was based on similarities regarding the distribution of observed and missing values over time, the observed trajectories in the intervention and control group, as well as the number of participants for each missing data pattern. Details about the categorization are outlined in the Results section. Generally, the formation of subgroups for pattern mixture models may depend on the type of study, pondering plausibility and statistical feasibility. For the present study, pattern mixture models were calculated with three subgroups: complete cases, participants with intermittent missing values, and participants who did not provide any follow-up data. To estimate the models, the KNOWNCLASS option in Mplus was used, creating a pseudo latent class variable. The overall model estimates were obtained by averaging the class-specific estimates, considering the proportion of each latent class in the total sample. Some parameters may be inestimable within one or more latent classes (e.g. the quadratic growth factor in a class where participants provided data in only two measurement occasions). This problem can be solved by parameter restrictions [21]. Three types of restrictions were implemented and compared. (i) For the complete case restriction, the inestimable parameters were fixed to the estimates of the complete case latent class (Mplus syntax 3e). (ii) For the neighbouring case restriction, the parameter of the most similar latent class was used (Mplus syntax 3f). (iii) The available case restriction replaces the inestimable parameters with the weighted average of the parameters in the other latent classes (Mplus syntax 3 g).

## Results

### Step 1: Missing data patterns and mechanisms in the PRINT trial

**Fig. 2:**
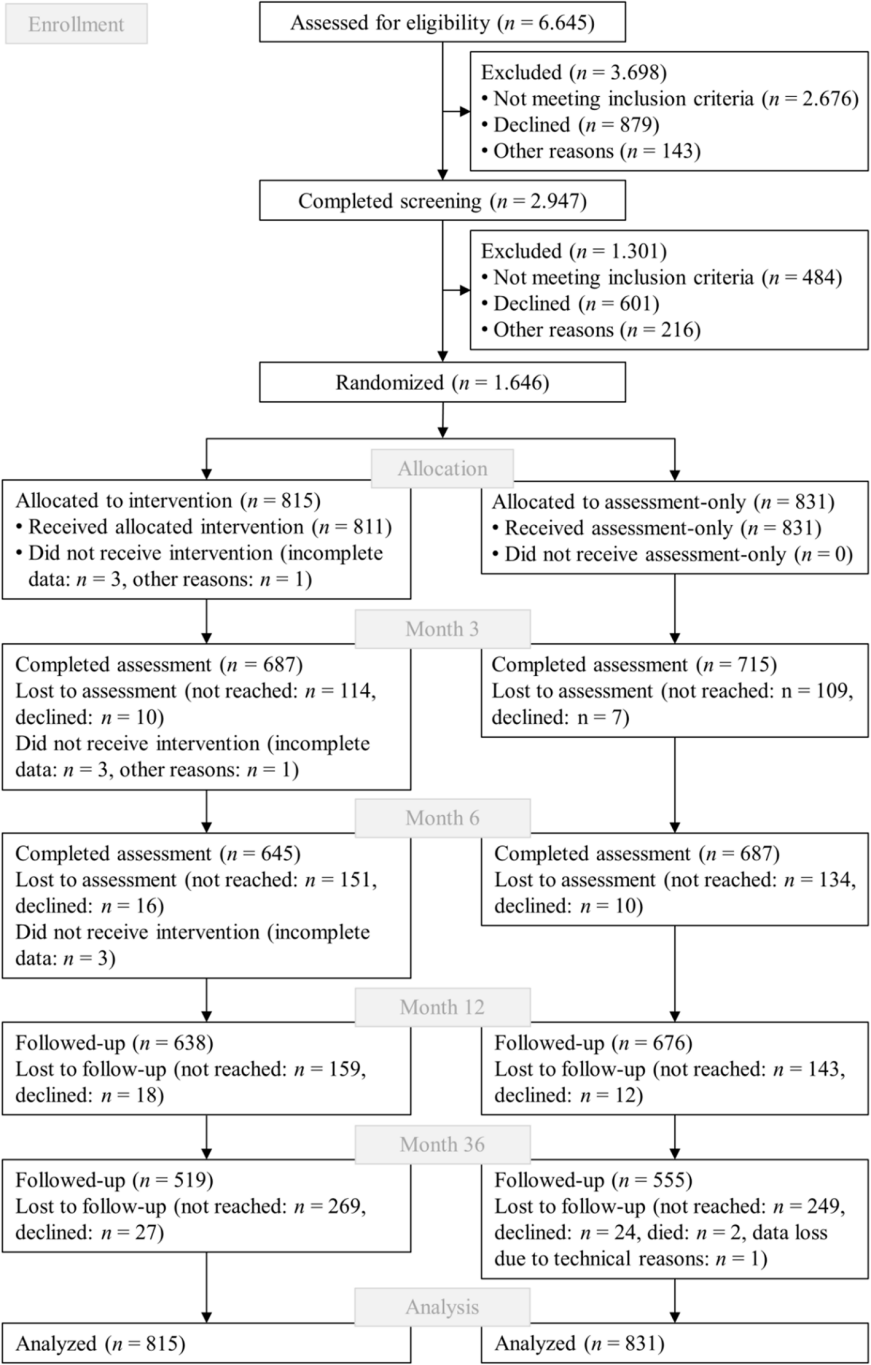
Flow of participants through the PRINT study

Of 1646 total participants (56% women; mean age = 31.0 ± 10.8 years), 80% (*n* = 1314) participated in the 12-month follow-up (t3) and 65% (*n* = 1074) in the 36-month follow-up (t4) assessment (Fig. 2).

Slightly higher participation rates were observed in the control compared to the intervention group (Table 3). Both groups reported lower AUDIT-C sum scores at t3 and t4 compared to baseline. The logistic regression models predicting participation at t3 and t4 revealed that older participants (*OR* = 1.04, *95% CI* = 1.02–1.05; and *OR* = 1.03, *95% CI* = 1.02–1.04) and those with 12 or more years of school education (*Ref.*: less than 12 years; *OR* = 2.05, *95% CI* = 1.55–2.73; and *OR* = 2.67, *95% CI* = 2.09–3.42) were more likely to participate. Smoking (*Ref.*: non-smokers; *OR* = 0.45, *95% CI* = 0.34–0.59; and *OR* = 0.49, *95% CI* = 0.39–0.63) and at-risk alcohol use at baseline (*Ref.*: low-risk alcohol use; *OR* = 0.76, *95% CI* = 0.59–0.99; and *OR* = 0.74, *95% CI* = 0.59–0.93) lowered the odds of providing data at t3 and t4, respectively. Thus, the propensity for outcome data at t3 and t4 to be missing can partly be explained by age, school education, smoking, and alcohol use at baseline, lending support to the assumption that the data are MAR.

**Table 3:** Observed AUDIT-C sum scores and proportions of observed and missing data

	Total sample		Intervention group		Control group	
	*M* (*SD*)	*n* (%) obs.	*M* (*SD*)	*n* (%) obs.	*M* (*SD*)	*n* (%) obs.
t0	3.51 (1.78)	1646 (100%)	3.49 (1.78)	815 (100%)	3.52 (1.79)	831 (100%)
t1	3.43 (1.87)	1407 (85%)	3.43 (1.86)	691 (85%)	3.43 (1.89)	716 (86%)
t2	3.20 (1.83)	1335 (81%)	3.20 (1.79)	648 (80%)	3.19 (1.87)	687 (83%)
t3	3.19 (1.90)	1.314 (80%)	3.17 (1.91)	638 (78%)	3.20 (1.90)	676 (81%)
t4	3.05 (2.02)	1074 (65%)	3.07 (1.98)	519 (64%)	3.04 (2.06)	555 (67%)

*obs.* Observed

**Fig. 3:**
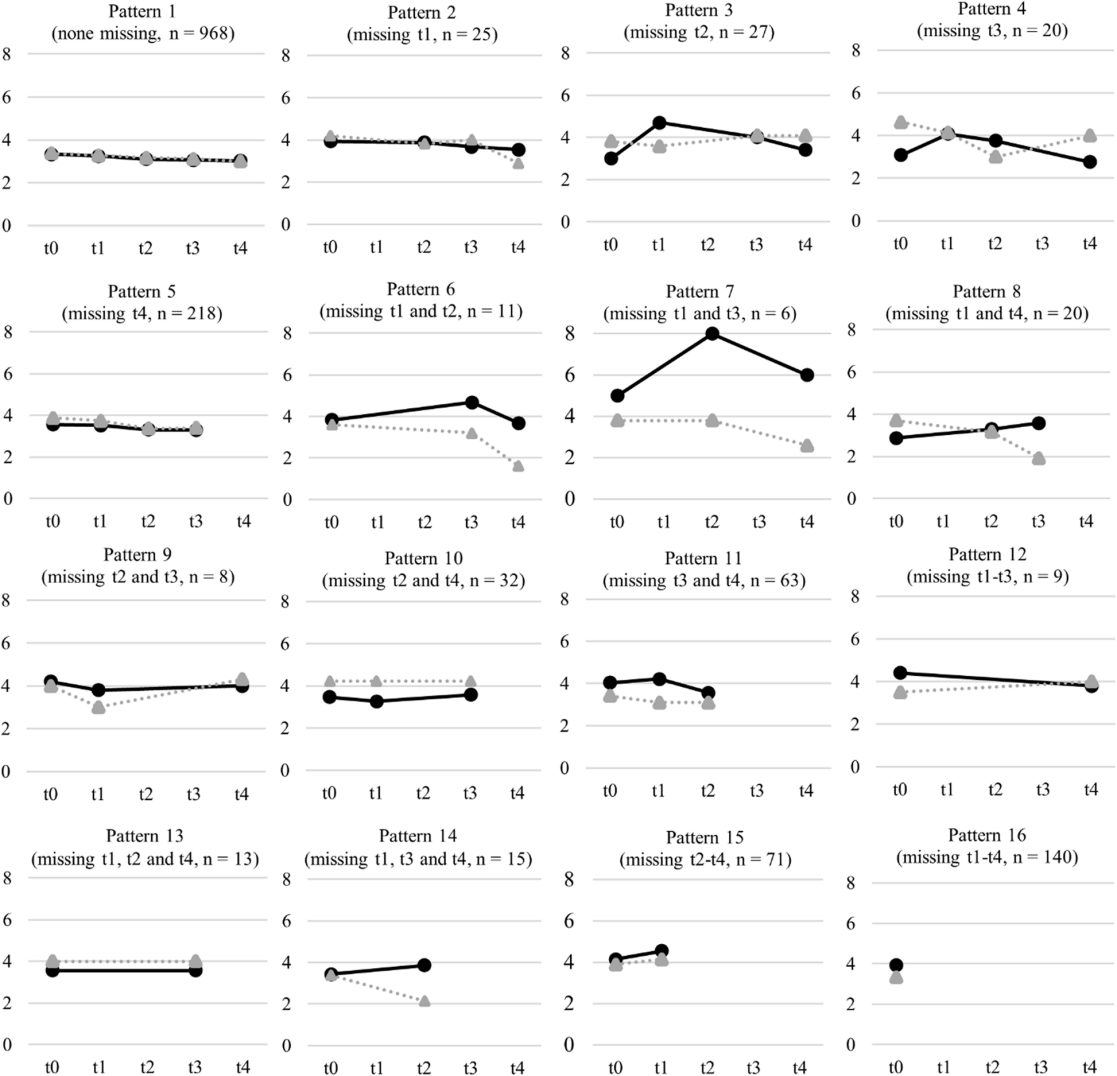
Observed AUDIT-C sum scores over time for each missing data pattern, separated by intervention (solid black lines) and control group (dotted grey lines)

The three most frequently observed missing data patterns (Fig. 3) were participants with no missing data (pattern 1, *n* = 968, 59%), participants with complete data except for t4 (pattern 5, *n* = 218, 13%), and participants with missing data at t1, t2, t3, and t4 (pattern 16, *n* = 140, 9%). Six missing data patterns showed similar trajectories, i.e. near-constant alcohol use over time (patterns 1, 2, 5, 9, 10, 12, and 13), representing 77% (*n* = 1273) of the sample. In some patterns, substantial temporal fluctuations were found (patterns 3, 4, 6, and 7), representing 4% (*n* = 64) of the sample. The trajectories observed in patterns 14 and 15 may suggest increasing alcohol use over time in participants who were lost at t3 and t4, representing 5% (*n* = 86) of the sample. Albeit only speculative, this might indicate a (missing) data generating process that is not at random, warranting sensitivity analyses.

### Step 2: MAR models

Neither the unadjusted nor the adjusted LGM provided evidence for an intervention effect. According to the unadjusted LGM, intervention group participants decreased their average AUDIT-C score from 3.50 at t0 to 3.27 at t3 and 3.18 at t4, respectively. A similar decrease was found in control group participants (from 3.50 at t0 to 3.18 at t3 and 3.10 at t4, respectively). Model-implied differences between intervention and control group were small in magnitude and not statistically significant (Table 4), except for the difference at t3 in the adjusted MAR model that suggested a higher AUDIT-C score in the intervention group.

**Table 4:** Model-implied differences in AUDIT-C scores between intervention and control group at t3 and t4 [95% confidence intervals]

	Group difference^a^		Difference in change^b^	
	t3^a^	t4^a^	t3^b^	t4^b^
Unadjusted MAR	0.087 [− 0.104; 0.278]	0.080 [− 0.144; 0.304]	− 0.088 [− 0.238; 0.062]	−0.081 [− 0.275; 0.113]
Adjusted MAR	0.178* [0.039; 0.316]	0.151 [−0.055; 0.358]	− 0.097 [− 0.231; 0.037]	−0.071 [− 0.261; 0.119]
DK (survival)	0.091 [− 0.100; 0.282]	0.085 [− 0.140; 0.310]	−0.092 [− 0.243; 0.059]	−0.085 [− 0.280; 0.109]
DK (multinomial)	0.090 [− 0.101; 0.281]	0.085 [− 0.140; 0.311]	−0.091 [− 0.241; 0.060]	−0.086 [− 0.281; 0.109]
WC (survival)	0.089 [− 0.088; 0.266]	0.086 [− 0.160; 0.332]	−0.089 [− 0.226; 0.048]	−0.086 [− 0.277; 0.105]
WC (multinomial)	0.038 [− 0.126; 0.203]	0.088 [− 0.158; 0.334]	−0.002 [− 0.026; 0.021]	−0.052 [− 0.240; 0.135]
PM (cc restriction)	0.111 [− 0.177; 0.400]	0.170 [− 0.581; 0.921]	−0.123 [− 0.393; 0.147]	−0.182 [− 0.932; 0.568]
PM (nc restriction)	0.090 [− 0.197; 0.378]	−0.117 [− 0.937; 0.703]	−0.103 [− 0.372; 0.167]	0.105 [− 0.717; 0.928]
PM (ac restriction)	0.109 [− 0.179; 0.398]	0.139 [− 0.612; 0.890]	−0.121 [− 0.391; 0.149]	−0.151 [− 0.901; 0.599]

*Abbreviations*: *DK* Diggle-Kenward selection model, *WC* Wu-Carroll shared parameter model, *PM* Pattern mixture model, *cc* complete case, *nc* neighbouring case, *ac* available case

* *p <* .05

^a^Positive values indicate higher AUDIT-C scores in the intervention group

^b^Positive values indicate a stronger decrease in AUDIT-C scores in the intervention group

### Step 3: MNAR models

The Diggle-Kenward selection models did not provide evidence for group differences over time (Table 4). The control group showed a marginally stronger decrease in their AUDIT-C scores over time compared to the intervention group, but the null was always included in the *95% CIs*. Notably, the logistic regressions predicting the missing indicators revealed no significant association between each AUDIT-C score and the propensity for that score to be missing when the missingness was coded with survival indicators (coefficient *log2* in Mplus syntax 3a). When multinomial missing indicators were used, higher AUDIT-C scores increased the probability of that score to be missing (coefficient *log2* in Mplus syntax 3b: *OR* = 1.22, *95% CI* = 1.08–1.38). Although based on untestable model assumptions, this finding suggested a missing data mechanism that is not at random.

A similar picture emerged for the Wu-Carroll shared parameter models. Control group participants tended to reduce their AUDIT-C scores slightly more than intervention group participants, but again the models yielded no statistically significant group differences (Table 4). The latent intercept predicted the missing indicators: higher initial AUDIT-C scores increased the probability of the subsequently assessed AUDIT-C scores to be missing. This was found both in the model with survival missing indicators (coefficient *log1* in Mplus syntax 3c: *OR* = 1.12, *95% CI* = 1.05–1.19) and the model with multinomial missing indicators (coefficients *log1* and *log3* in Mplus syntax 3d: *OR* = 1.18, *95% CI* = 1.07–1.29 and *OR* = 1.13, *95% CI* = 1.04–1.24) and is consistent with the assumed MAR mechanism based on the prediction of follow-up participation. The latent slope neither predicted the survival nor the multinomial missing indicators. Thus, the Wu-Carroll models provided no indication that the missing values were MNAR.

For the pattern mixture approach, the 16 missing data patterns (Fig. 3) were condensed into three groups. This decision was mainly based on similarities regarding participation and non-participation over the course of the trial as well as the statistical feasibility of the resulting sample sizes in those subgroups. *Complete cases* (73%, *n* = 1206; missing data patterns 1, 4, and 5) received the intervention as planned in the study protocol and provided data at least at one follow-up assessment. *Participants with intermittent missing values* (9%, *n* = 151; missing data patterns 2, 3, 6–10, 12, and 13) did not receive the full intervention as these participants missed at least one assessment during the active intervention phase but provided follow-up data at least once. The third group were *participants who did not provide any follow-up data* (18%, *n* = 289; missing data patterns 11, 14–16). No statistically significant group differences were found in any of the pattern mixture models (Table 4). If anything, control group participants tended to reduce their AUDIT-C scores slightly more than intervention group participants but again, the null was always included in the *95% CIs*. As the modelled trajectories over time were very similar between the latent classes in each of the three pattern mixture models, there was no indication to support the MNAR assumption.

## Discussion

The aim of the present paper was to demonstrate sensitivity analyses for different assumptions regarding the missing data mechanism for randomised controlled trials. For that purpose, using data from a brief alcohol intervention trial, latent growth models were estimated that either assumed the missing data to be MAR or MNAR. There was no difference in the change of alcohol use over time between intervention and control group. No clear evidence for intervention effects on the AUDIT-C score emerged. The analytical approach illustrated in this study allowed us to ascertain that our findings were insensitive to different missing data mechanisms.

In randomised controlled trials, attrition and missing data will most likely occur. One can never determine with certainty if missing values are MAR or MNAR [5]. In any case, researchers have to contemplate how to handle missing data in order to prevent bias and false conclusions from improperly handled missingness [14, 15]. To facilitate an easy to implement missing data strategy, our aim was to shed light on a sensitivity approach using LGM that can be roughly divided into three main steps. First, information about the potential data generating process was gathered. Examining the degree of missing data, inspecting all possible missing data patterns, and predicting follow-up participation with baseline variables allowed us to accumulate evidence for the missing data mechanism. Second, unadjusted and adjusted growth models were calculated to analyse intervention effects over time. These models were based on an MAR assumption and applied FIML estimation, known to produce unbiased parameter estimates when data are truly missing at random [12]. Third, the findings from step two were safeguarded by incorporating an MNAR mechanism into the growth model. To that end, we illustrated different versions of selection [28], shared parameter [29] and pattern mixture models [30]. Model comparisons enabled us to assess how sensitive our findings were to different assumptions about the missing data mechanism (MAR versus MNAR). The distinction between MAR and MNAR still remained speculative but the main finding about the intervention’s efficacy was corroborated by different models that all arrived at the same conclusion: we found no evidence for intervention efficacy after 36 months.

Missing data in longitudinal studies are virtually unavoidable. Therefore, a missing data strategy is already needed in the planning stage of randomised controlled trials. Considerations should not only address how to prevent dropout and keep participants engaged within the study protocol, but also how to deal with missing values in statistical analyses. The latter also involves contemplating on variables that may be associated with participant attrition. Currently, information for the explanation of missingness are mostly selected post hoc, using readily available variables. If variable selection is already systematically thought about during planning, the analysis of missing data will be more informative. In this respect, process data can also be very useful, such as the number of contact attempts needed to reach someone per phone, or the type of contact information someone provides when giving their informed consent (landline or mobile phone number, e-mail address, or both). Beyond that, addressing the issue of missing data early on would help to better account for reach as one important dimension of public health intervention success [46].

Conducting sensitivity analyses applying LGM has several strengths. First, growth modelling is a flexible tool to analyse inter-individual differences in intra-individual trajectories over time [23]. Considerations about missing data mechanisms may depend on topic, study design and procedures, sample composition, as well as reach and retention rates. Growth modelling is a customisable framework that enables researchers to take all these aspects into account and gather evidence on why data are missing. Second, the outlined maximum likelihood approach follows an intention-to-treat principle and ensures unbiased estimates and sufficient statistical power against the backdrop of missing data [12, 47]. Thus, the conclusions drawn from LGMs are superior in validity compared to still widely used, but disadvantageous strategies such as complete case analysis and single imputation methods [9, 11]. Third, growth modelling is already starting to be used in the evaluation of randomised controlled health behaviour trials [25, 26, 48–51]. Hence, additional sensitivity analyses for the missing data to be MNAR may be done without great additional effort. Fourth, the approach is able to provide nuanced insight into how conclusions about intervention effects change depending on the assumed mechanism of participant attrition. Consistent findings as in our case may underpin the initial conclusions about an intervention’s efficacy or non-efficacy. Inconsistent findings on the other hand would suggest that conclusions based on a single missing data mechanism (most likely MAR) may be flawed. In this case, an intervention’s efficacy may be subject to who completed the study and who did not, certainly making the interpretation of findings more complex (for examples see [21, 52, 53]), but also highlighting the need to better understand the reasons for study dropout and to optimise an intervention with respect to participant retention.

Yet, limitations have to be acknowledged. All illustrated MNAR models are based on untestable model assumptions. Violations of these assumptions may lead to biased parameter estimates [21]. The shared parameter models were computationally demanding, as they required Monte Carlo integration. What is more, the models have only been demonstrated with a continuous outcome. In practice, primary endpoints may be count, categorical or dichotomous. Although LGM can be applied with non-continuous repeated outcome variables [54–56], implementing the MNAR extensions in these models may be challenging and complicate successful model convergence. Our approach did not explain the mathematical or technical grounds. Interested readers may consider seminal work [6, 21, 28, 29] outlining the technical details of the models.

Sensitivity analyses are widely recommended to be included in the statistical repertoire in the evaluation of intervention studies [17, 20, 21, 33–35]. The approach we sketched out in this paper is just one way to put this recommendation into practice, in particular for those familiar with LGM. Other approaches should not go unmentioned. For instance, research has suggested distinguishing different subtypes of the MNAR missing data mechanism [57]. Sensitivity analyses may also be implemented using multiple imputation [58–60] or Bayesian statistics [61–63].

## Conclusions

Participant attrition and dropout is ubiquitous in empirical intervention studies, and so is the need for researchers to reflect on how to handle missing values. Still, missing data mechanisms are not examined rigorously [7–9, 36]. To aid in closing that gap, our aim was to demonstrate a comprehensible and straightforward maximum likelihood estimation approach to determine the sensitivity of intervention efficacy findings for two cases: when data are missing at random and when data are not missing at random. For that purpose, we provided instructions and Mplus syntax for MAR and MNAR growth models, namely Diggle-Kenward selection [28], Wu-Carroll shared parameter [29] as well as pattern mixture models [21]. This may encourage researchers to conduct sensitivity analyses for different missing data mechanisms in order to safeguard findings from randomised controlled trials against the pitfalls of (non-ignorable) missing data.

## Data Availability

The dataset and syntax supporting the conclusions of this article are available via the Research Data Centre at Leibniz Institute for Psychology (ZPID) and can be accessed via 10.5160/psychdata.stas21pr11.

## Abbreviations

AUDIT-C: Alcohol Use Disorders Identification Test – Consumption
CI: Confidence interval
FIML: Full-information maximum likelihood
IRR: Incidence Rate Ratio
LGM: Latent growth model
MAR: Missing at random
MCAR: Missing completely at random
MNAR: Missing not at random
OR: Odds Ratio
PRINT: Randomized controlled trial “Testing a proactive expert system intervention to prevent and to quit at-risk alcohol use”
